# Double blind randomized crossover trial of PF-03654764+fexofenadine in the environmental exposure unit (EEU)

**DOI:** 10.1186/1710-1492-10-S1-A68

**Published:** 2014-03-03

**Authors:** Michelle L North, Terry Walker, Lisa M Steacy, Barnaby G Hobsbawn, Richard J Allan, Frances Hackman, Xiaoqun Sun, Andrew G Day, Anne K Ellis

**Affiliations:** 1 Department of Biomedical and Molecular Sciences, Queen’s University, Kingston, Ontario, Canada, K7L 2V5; 2 Allergy Research Unit, Kingston General Hospital, Kingston, Ontario, Canada, K7L 2V7; 3 Pfizer Ltd., Sandwich, Kent, CT13 9NJ, UK; 4 Clinical Research Centre, Kingston General Hospital, Kingston, Ontario, Canada, K7L 2V7; 5 Division of Allergy and Immunology, Department of Medicine, Queen’s University, Kingston, Ontario, Canada, K7L 2V7

## Background

Oral histamine receptor–1 antagonists, such as fexofenadine, offer suboptimal relief of allergic rhinitis-associated nasal congestion. Combinations with oral sympathomimetics, such as pseudoephedrine, relieve congestion but produce side effects. Histamine receptor-3 antagonists, such as PF-03654764, reduce congestion in animals and have been proposed as novel therapeutics. Previous nasal allergen challenge studies of similar H1+H3 receptor antagonist combinations demonstrated reduced congestion. Herein we employ the Environmental Exposure Unit (EEU) to conduct the first randomized controlled trial of PF-03654764 in allergic rhinitis. The primary objective was to compare the effect of PF-03654764+fexofenadine to pseudoephedrine+fexofenadine on the subjective measures of congestion and Total Nasal Symptom Score (TNSS). The objective of post-hoc analyses were to compare all treatments to placebo and determine the onset of action (OA).

## Methods

64 participants were randomized in a double-blind, placebo-controlled 4-period crossover study. Participants were exposed to ragweed pollen for 6 hours post-dose in the EEU.

## Results

PF-03654764+fexofenadine was not superior to pseudoephedrine+fexofenadine. In post-hoc analyses, PF-03654764+fexofenadine significantly reduced TNSS, relative to placebo, and OA was 60 minutes. Pseudoephedrine+fexofenadine significantly reduced congestion and TNSS, relative to placebo, with OA of 60 and 30 minutes, respectively. All PF-03654764-treated groups experienced an elevated incidence of adverse events.

## Conclusions

PF-03654764+fexofenadine failed to provide superior relief of allergic rhinitis-associated nasal symptoms upon exposure to ragweed pollen compared to fexofenadine+pseudoephedrine. However, PF-03654764+fexofenadine improved TNSS compared to placebo. Side effects were not insignificant.

